# Treatment with Botulinum toxin A in a total population of children with cerebral palsy - a retrospective cohort registry study

**DOI:** 10.1186/s12891-017-1880-y

**Published:** 2017-12-11

**Authors:** Maria Franzén, Gunnar Hägglund, Ann Alriksson-Schmidt

**Affiliations:** 1 Faculty of Medicine, Lund University, Lund, Sweden; 2 Department of Clinical Sciences Lund, Orthopedics, Skåne University Hospital, Lund University, Lund, Sweden

**Keywords:** Cerebral palsy, Spasticity, Botulinum toxin a, CPUP, Age, Sex, GMFCS level, Registry

## Abstract

**Background:**

Botulinum toxin A (BTX-A) has been used to reduce spasticity in children with cerebral palsy (CP) for decades. The purpose of this study was to analyze to what extent BTX-A treatment was used to treat spasticity in a total population of children with CP. We investigated 1) the use of BTX-A in relation to age, sex, and Gross Motor Function Classification System (GMFCS) level, 2) the most common muscle groups treated with BTX-A in relation to the same variables, and 3) changes in the proportions of children treated with BTX-A between two time points (2010 and 2015).

**Methods:**

The study was based on data from CPUP, a combined Swedish follow-up program and national healthcare registry, comprising >95% of all children with CP in Sweden. The participants (*N* = 3028) were born in 2000 or later. Potential BTX-A treatment and treated muscle groups were included from all CPUP assessments recorded in the registry in 2014–2015. In Aim 3, BTX-A administration in 3–5 year-olds at two time points was assessed. Crosstabs and 95% confidence intervals (CIs) for binominal proportions were calculated and logistic regression was used to regress age, sex, and GMFCS level on BTX-A treatment. Muscle groups treated with BTX-A were assessed using crosstabs and 95% CIs. Proportional change in BTX-A treatment over a 5-year period was analyzed using chi-square.

**Results:**

We included 3028 children (57% boys; median age 7 years) of whom 26% received BTX-A. Significantly more boys (28%) than girls (23%) received BTX-A (OR = 1.25, [95% CI 1.05–1.48]). Significant differences were found for age and GMFCS levels; 4–6 year-olds and those at GMFCS III-IV were more likely to receive BTX-A. BTX-A treatment in the gastrocnemius muscle was most common in the 4–6 year-olds and at GMFCS I-III, whereas treatment of the hamstring and adductor muscles was more common in older children and at GMFCS IV-V. No significant change in the proportion of BTX-A administered in 2010 and 2015 was demonstrated.

**Conclusions:**

BTX-A treatment differed based on age, sex, and GMFCS level. Proportion of BTX-A treatment in Sweden has remained stable during the past five years.

**Electronic supplementary material:**

The online version of this article (10.1186/s12891-017-1880-y) contains supplementary material, which is available to authorized users.

## Background

Cerebral palsy (CP) is the most common musculoskeletal disability in childhood; the reported prevalence is 2.0–3.0 per 1000 live births [1, 2]. Spasticity is a dominant symptom in many individuals with CP and a component of the upper motor neuron syndrome. It refers to a velocity-dependent abnormally high muscle tone (hypertonia) resulting from hyper excitability of the stretch reflex [3]. Although the spasticity may have a positive effect in some children with CP, due to compensation for muscle weakness, it can also inhibit mobility control, function, and activity [4]. The musculoskeletal development may also be affected by the increased muscle tone, leading to muscle shortening, torsional deformities, hip dislocation, and scoliosis [4, 5].

Since 1993, intramuscular injections with Botulinum Toxin A (BTX-A) have been used to reduce spasticity in individuals with CP [6]. The toxin produces a local paralysis by blocking the presynaptic release of the neurotransmitter acetylcholine in the neuromuscular junction [7] and, thereby, reduces the hyperactivity and spasticity in the muscle. The chemical denervation of BTX-A is reversible and consequently treatment normally only has a temporary effect, lasting for three to four months [8, 9]. Although BTX-A has been used to treat spasticity for over 20 years it is still partly considered an unexplored area. Opinions differ regarding the indications for BTX-A treatment and its long-term effect and use have been sparsely investigated [10, 11]. Moreover, little is known about the use of BTX-A in the population of children with CP. This information could facilitate the development of national guidelines.

In this study, we investigated to what extent BTX-A treatment was used, and the most common muscle groups treated with BTX-A, in a total population of children with CP living in Sweden. The specific aims were to analyze (1) the proportion of children with CP in Sweden treated with BTX-A in relation to age, sex, and Gross Motor Function Classification System (GMFCS) level, (2) the most common muscle groups treated in relation to the same variables listed in Aim 1 and, (3) changes in the proportion of children with CP treated with BTX-A at two time points (2010 and 2015).

## Methods

This was a retrospective cohort registry study using data from CPUP from 2009 to 2010 and 2014–2015.

### Procedure

**Fig. 1:**
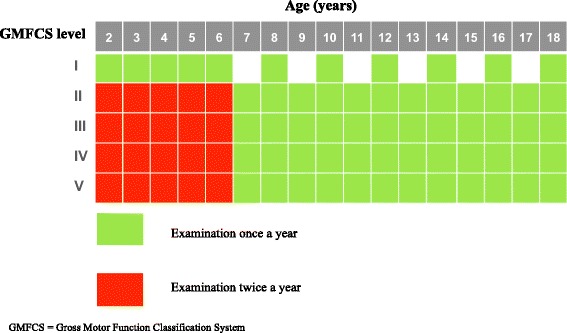
Assessment schedule for CPUP

CPUP is a combined follow-up program and national healthcare registry for people with CP in Sweden. The registry encompasses all 21 healthcare regions in Sweden and includes more than 95% of the children with CP in Sweden [12]. Families of a child with CP are informed about CPUP and have the option of not participating in the registry, a choice that will not affect the child’s healthcare received. When choosing to participate in CPUP, the families are verbally and in writing informed that data recorded in the registry may be used for research and quality improvement projects. They are also informed that the data will be handled and presented in a non-identifiable manner in accordance with current legislation and that they, at any time, may withdraw their participation and if choosing to do so the data in the registry will be deleted. This information is available at CPUP’s website [13]. The Ethics Committee has approved the use of verbal consent. The CP diagnosis, based on the definition established by the Surveillance of Cerebral Palsy Network in Europe (SCPE), is determined by a pediatric neurologist at the age of four years [14]. This means that a small number of infants and toddlers who eventually may be found not to have CP are followed according to the CPUP protocol during the first four years of life, and are thus included in the CPUP database (removed once diagnoses have been changed). The GMFCS is a categorization system based on the children’s self-initiated movement with focus on sitting, walking, and mobility. An ordinal scale from I to V is used, where level I indicates the least and level V the most affected gross motor function. It has been demonstrated that the individual GMFCS level in most children with CP remains stable over time [15], although there are exceptions [16]. All GMFCS levels are represented in the CPUP registry. The children are regularly examined by their local physical therapists (PTs) and occupational therapists (OTs) in a standardized manner, according to a specific CPUP protocol [17, 18]. Gross motor function, hand function, mobility, range of joint motion, postural ability in standing, sitting and lying, muscle spasticity, pain and pain sites, and if the child has received BTX-A since the last CPUP assessment are examples of what are examined at every CPUP assessment. The results from the examination are recorded in the registry after each CPUP assessment. The time interval between two clinical examinations varies depending on the age of the child and the GMFCS level (Fig. 1).

### Participants

Participants in CPUP born 2000 or later were eligible for inclusion. Those who had undergone selective dorsal rhizotomy (SDR) or had an intrathecal baclofen (ITB) pump were excluded (*n* = 91). Three participant cohorts were constructed referred to as Cohort 1, Cohort 2, and Cohort 3 hereon after. Cohort 1 was used in Aims 1 and 2 to analyze the relationship between BTX-A treatment in relation to age, sex, GMFCS level, and muscle groups treated. Cohort 1 consisted of the total number of 3028 children aged 1–15 years, recorded in the registry in 2014–2015. Cohorts 2 and 3 were used in Aim 3 to investigate changes in BTX-A treatment between two time periods. Cohort 2 consisted of children three to five years of age, recorded in the registry 2014–2015 (*n* = 736) and Cohort 3 consisted of children of the same ages, recorded 2009–2010 (*n* = 649). The age-span three to five years was chosen to create two independent groups, in order that a participant would only be represented in one group.

**Fig. 2:**
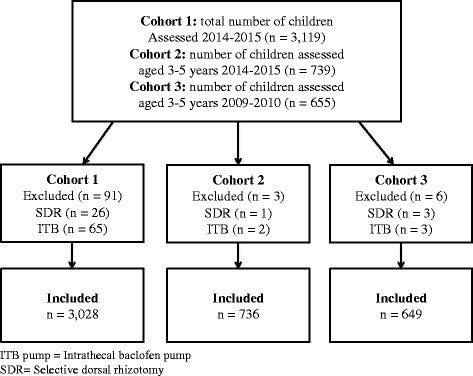
Flowchart of study inclusion

Figure 2 illustrates how the three cohorts were assembled according to the exclusion criteria.

In total, 3028 children with a median age of 7 years (SD = 4 years), ranging from 1 to 15 years were included in Cohort 1. The age, sex, and distributions of GMFCS levels in Cohort 1 are presented in Table 1.

**Table 1:** Distribution of age, sex, and GMFCS level (Cohort 1)

	Boys *n* (%)	Girls *n* (%)	Total *n* (%)
Age (years)			
1–3	334 (19)	279 (21)	613 (20)
4–6	416 (24)	287 (22)	703 (23)
7–9	393 (23)	270 (21)	663 (22)
10–12	371 (22)	291 (22)	662 (22)
13–15	211 (12)	176 (14)	387 (13)
GMFCS level			
I	748 (43)	589 (45)	1337 (44)
II	310 (18)	206 (16)	516 (17)
III	165 (10)	118 (9)	283 (10)
IV	263 (15)	197 (15)	460 (15)
V	239 (14)	193 (15)	432 (14)
Total	1725 (57)	1303 (43)	3028 (100)

Number of children, *n* (%), in relation to age, sex, and GMFCS level in Cohort 1.

GMFCS = Gross Motor Function Classification System.

### Measures

Data on age, sex, and GMFCS-level were extracted from the CPUP database. Age was calculated based on the date of birth and the date of examination, then rounded to whole years, grouped into intervals of three years, and treated as a categorical variable. The gross motor function was classified according to the extended and revised version of the GMFCS [19]. In this study, we did not take into account whether or not the GMFCS level changed for the children between the CPUP assessments. In children with more than one assessment during the time period of interest, age, sex, and the GMFCS-level reported at the first assessment was used. For all aims (all cohorts), BTX-treatment was coded as yes if the participants had received BTX-A at any CPUP assessment during 2009–2010 or 2014–2015, and no if the participant had not received BTX-A at any of the CPUP assessments. If participants had missing data on this specific item they were subsequently analyzed as not having received BTX-A treatment.

The muscle groups treated with BTX-A were categorized as the gastrocnemius, the hamstring, or the adductor muscles in total (i.e., the total amount of treatments in one muscle), single (i.e., when only one muscle was treated at the time) or in combination (i.e., two or more of the muscles above were treated at the same time). Additional muscles and other combinations of muscles treated were combined into one group. Missing data also included children who received BTX-A but who were missing the information about what muscle was injected. The distribution of muscle injections is presented in Table 2. Additional muscles and muscle combinations treated are available in Additional file 1.

**Table 2:** Distribution of children treated with BTX-A in different muscle groups (Cohort 1)

Muscle injected	Total *N* (%)
Gastrocnemius total	590 (76)
Hamstrings total	321 (41)
Adductors total	231 (30)
Gastrocnemius alone	266 (34)
Hamstrings alone	38 (5)
Adductors alone	23 (3)
Gastrocnemius + Hamstrings	99 (13)
Gastrocnemius + Adductors	40 (5)
Hamstrings + Adductors	49 (6)
Gastrocnemius + Hamstrings + Adductors	53 (7)
Other combinations^a^ (see Additional file 1)	183 (24)
Missing data^b^	25 (3)
Total number of children treated	776 (100)

BTX-A = Botulinum Toxin A

^a^Other combinations include additional muscles to those mentioned here, alone or in combination with gastrocnemius, hamstrings, and adductors muscles, and are presented in Additional file 1

^b^Data missing on what muscle was treated with BTX-A

The study was approved by the Ethics Board at Lund University (LU 443–99, revised 2009).

### Data analysis

Distributions of the data were inspected and continuous variables were presented as medians and standard deviations (SD), and categorical variables as frequencies (n) and percentages (%). For Aim 1, the proportion of BTX-A treatment in relation to age, sex, and GMFCS level were analyzed using cross-tabs and 95% confidence intervals (CIs) for binomial proportions and logistic regression (odds ratios, OR). Both main effects (age, sex, and GMFCS levels) and interactions (age X sex; age X GMFCS level; sex X GMFCS level) were tested for in the logistic regression. For Aim 2, the relationships between the three different muscle groups (total injections) treated with BTX-A (%) in relation to age, sex, and GMFCS level were calculated using cross-tabs and 95% CIs for binomial proportions. In Aim 3, chi-square tests (*χ* ^2^) were used to compare differences in use of BTX-A between 2010 and 2015. IBM SPPS version 22 [20] and Stata 13 [21] were used for all analyses.

## Results

**Fig. 3:**
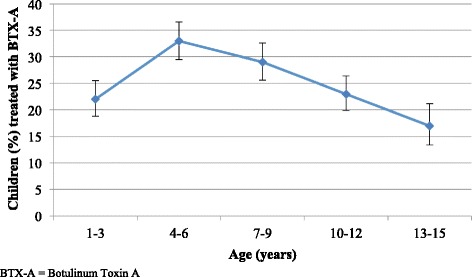
BTX-A treatment in relation to age. Proportion of children (%) treated with BTX-A based on age. The line segments represent the upper and lower bounds of the 95% confidence intervals. *N* = 3028

**Fig. 4:**
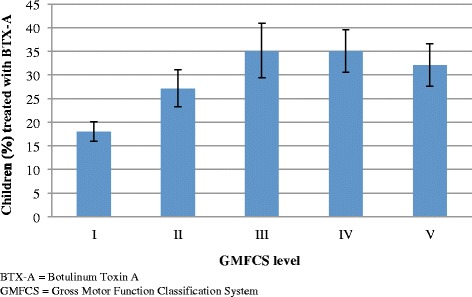
BTX-A treatment in relation to GMFCS level. Proportion of children (%) treated with BTX-A based on GMFCS level. The line segments represent the upper and lower bounds of the 95% confidence intervals. N = 3028

In Cohort 1, 776 children (26%) were reported to have received BTX-A treatment at least once since the previous CPUP assessment. The proportion of children treated with BTX-A varied with age (Fig. 3), peaking at the age of four to six years. Boys were statistically significantly more likely to have received BTX-A treatment (28%, [95% CI 25.5–29.8]) than girls (23%, [95% CI 20.8–25.4]). BTX-A treatment also varied with GMFCS level (Fig. 4).

The ORs and 95% CIs are presented in Table 3. Interactions were not statistically significant and are not presented.

**Table 3:** BTX-A treatment related to age, sex, and GMFCS level

Variable (reference group)	Odds ratio (95% CIs)
Age (1–3 years of age)	
4–6 years of age	2.02 (1.57–2.61)
7–9 years of age	1.53 (1.18–1.99)
10–12 years of age	1.14 (0.87–1.50)
13–15 years of age	0.81 (0.58–1.13)
Sex (Girls)	
Boys	1.25 (1.05–1.48)
GMFCS level (Level I)	
Level II	1.75 (1.37–2.24)
Level III	2.59 (1.94–3.45)
Level IV	2.58 (2.02–3.28)
Level V	2.27 (1.77–2.92)

The odds ratio and 95% CIs of children treated with BTX-A in relation to age, sex, and GMFCS level.

BTX-A = Botulinum Toxin A.

CIs = confidence intervals.

GMFCS = Gross Motor Function Classification System.

**Fig. 5:**
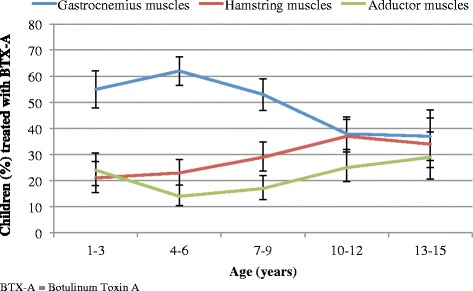
BTX-A treatment based on muscle groups and age. Proportion of children (%) treated with BTX-A based on muscle groups and age. The line segments represent the upper and lower bounds of the 95% confidence intervals. *N* = 776

Data on muscle groups treated with BTX-A were available for 751 of the 776 children (97%) who received BTX-A in Cohort 1. In all age categories the gastrocnemius muscle was the most frequently muscle treated, particularly in children one to nine years of age. Injections in the hamstring and adductor muscles were more common at older ages (10–15 year-olds) compared to in younger children. The most common muscle groups treated with BTX-A in relation to age are presented in Fig. 5.

**Fig. 6:**
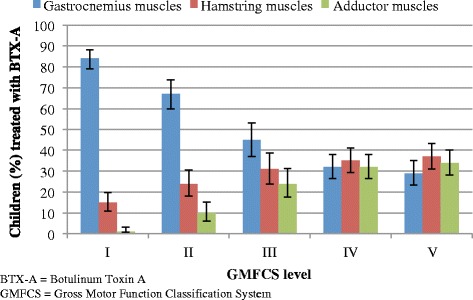
BTX-A treatment based on muscle groups and GMFCS level. Proportion of children (%) treated with BTX-A based on muscle groups and GMFCS level. The line segments represent the upper and lower bounds of the 95% confidence intervals. *N* = 776

Children at GMFCS I-III most often received BTX-A treatment in the gastrocnemius muscle. Children at GMFCS IV-V were more likely than the other GMFCS categories to receive BTX-A treatment in the hamstring and adductor muscles (Fig. 6). The proportion of children treated with BTX-A in the gastrocnemius muscle decreased with GMFCS level (Fig. 6).

A total of 248 children (34%) in Cohort 2 and 228 children (35%) in Cohort 3 had received BTX-A at least once since the previous CPUP assessment. No statistical significant differences were found in the proportion of BTX-A treatment between 2009 and 2010 and 2014–2015 as shown by chi square test, χ^2^ (1, *N* = 1385) = 0.32, *p* = .575.

## Discussion

This study was based on a total population of children with CP in Sweden aged one to fifteen years. Approximately one fourth of all children with CP had received BTX-A treatment at least once since the previous CPUP assessment. The use of BTX-A varied with age, sex, and gross motor function.

BTX-A treatment was most often used in the four to six year-olds. This corresponds to a previous study describing the development of spasticity of the gastrocnemius muscle where an increase in muscle tone was found up to four to six years of age, then followed by a gradual reduction in muscle tone [22]. Our results thus indicate that most children receive BTX-A treatment when they are likely to have the highest degree of spasticity.

Boys were significantly more likely to be treated with BTX-A than girls. Although a small difference, this has, to our knowledge, not been demonstrated previously. More boys than girls have CP [2, 23], yet the distribution of the severity of CP, in terms of gross motor function and CP-subtypes, has not been found to differ significantly between the sexes [2, 24]. One explanation for this sex difference might be that the degree of spasticity differs between boys and girls. However, an alternative explanation might be a bias of the treating provider, or the parents of the child, that boys are expected to be more physically active than girls and that they therefore are more likely to treat boys than girls with BTX-A. This hypothesis is supported by a recent study, also based on CPUP data, showing that boys received more physical therapy than girls [25].

Children at GMFCS levels II to V were more often treated with BTX-A compared to children at GMFCS level I. Most children with unilateral spastic CP are at GMFCS level I and generally have lower degrees of spasticity [22]. Children at higher GMFCS levels have a higher degree of spasticity and might therefore be at an increased risk of developing muscle contractures, which is in line with higher proportions of BTX-A treatments in these children.

Overall, the gastrocnemius muscle was the most commonly muscle treated with BTX-A, especially in the younger children and in children at GMFCS levels I-III. This has been reported previously and might be related to the fact that equinus foot is the most common deformity in children with CP [26]. Also, the development of contractures of the gastrocnemius muscle has been found to be greatest at younger ages and at GMFCS I and II [27]. The hamstring and adductor muscles were more often treated in older children (13 to 15-year olds) and in children at GMFCS levels IV-V. This might be because the development of muscle contractures is highest in the hamstring and adductors muscles at these ages, and at GMFCS IV and V [27].

The proportion of children treated with BTX-A did not change between 2009 and 10 and 2014–15. This indicates that although some authors in recent years have favored a more restrictive approach of BTX-A use [10, 28], the quantity of BTX-A treatment has not been affected in Sweden the past five years. However, we do not know if there was a change in the use of BTX-A prior to 2010.

There were a number of limitations to this study. The classification of subtype was missing in several cases and therefore not included in the analyses. If the information of subtype had been available, a better understanding of the results regarding different muscle groups treated might have been possible. When performing secondary analyses, the investigator is limited to the variables included in the registry. Moreover, registers, by necessity, cannot include too vast a number of varibles because that might compromise compliance, and, by extension, coverage rates. Additional variables that would have been interesting to analyze would have been if one or both legs were treated at the same time and what dose of BTX-A was used. On the other hand, what makes registry studies so powerful is the coverage rate, in the case of CPUP virtually the total population, which strengthens generalizability and allows for external validity. By using data from the CPUP we were able to include all GMFCS levels, even those children less likely to receive BTX-A, which provided an unselective material and thereby reduced the risk of selection bias.

This study was the first step in identifying the distribution of BTX-A treatment in a total population of children with CP and demonstrated a variation in administration of BTX-A, in relation to age, sex, GMFCS level, and muscle groups treated. To date, no evidence-based recommendations or guidelines are available in terms of BTX-A treatment in children and adolescents living with CP. In order to initiate work that could eventually result in such recommendations, it is important to know what the current practice looks like. This study provides that kind of information based on the total population in one entire country.

## Conclusion

Treatment with BTX-A in Sweden varied in relation to age, sex, and GMFCS level. Muscle groups treated also varied with age and GMFCS level and corresponded to the known development of spasticity and muscle contractures. The proportion of BTX-A treatments given has not changed over the past five years.

## Additional file

Additional file 1: Combinations of muscles injected with BTX. Every combination of muscles treated with Botulinum Toxin A (BTX-A). Missing data = received BTX-A, but without the information on what muscle treated. (PDF 63 kb)

## Abbreviations

BTX-A: Botulinum Toxin A
CP: Cerebral Palsy
CPUP: Swedish Cerebral Palsy Surveillance Program and National Healthcare Registry
GMFCS: Gross Motor Function Classification System
ITB pump: Intrathecal Baclofen Pump
SCPE: Surveillance of Cerebral Palsy Network in Europe
SDR: Selective Dorsal Rhizotomy
