# Supplementary material for: Treatment with Botulinum toxin A in a total population of children with cerebral palsy - a retrospective cohort registry study
Source: BMC Musculoskelet Disord. 2017 Dec 11;18:520. doi: 10.1186/s12891-017-1880-y (PMC5725838; doi:10.1186/s12891-017-1880-y)
Supplement: Additional file 1: — Combinations of muscles injected with BTX. Every combination of muscles treated with Botulinum Toxin A (BTX-A). Missing data = received BTX-A, but without the information on what muscle treated. (PDF 63 kb) [file 12891_2017_1880_MOESM1_ESM.pdf]

**Combination of muscles injected with BTX-A**	**Number of treatments (n=776)**
gastrocnemius	266
gastrocnemius+hamstrings	99
gastrocnemius+hamstrings+adductors	53
gastrocnemius+tibialis posterior	52
hamstrings+adductors	49
gastrocnemius+adductors	40
hamstrings	38
adductors	23
gastrocnemius+hamstrings+gracilis	8
gastrocnemius+hamstrings+tibialis posterior	8
hamstrings+adductors +psoas major	7
gastrocnemius+hamstrings+adductors+upper extremity	6
gastrocnemius+hamstrings+adductors+tibialis posterior	5
gastrocnemius+upper extremity,	5
gastrocnemius+adductors+psoas major	4
gastrocnemius+hamstrings+adductors+psoas major	4
hamstrings+adductors+rectus femoris	4
gastrocnemius+adductors+upper extremity	4
hamstrings+gluteus maximus	3
gastrocnemius+gracilis	3
psoas major	3
adductors+rectus femoris	3
hamstrings+adductors+rectus femoris	3
gastrocnemius+hamstrings+upper extremity	3
hamstrings+adductors+upper extremity+rectus femoris	3
hamstring+gracilis	2
gastrocnemius+hamstrings+psoas major	2
gastrocnemius+rectus femoris	2
gastrocnemius+hamstrings+adductors+rectus femoris+psoas major	2
adductors+rectus femoris	2
tibialis posterior	2
gastrocnemius+adductors+tibialis posterior	2
hamstrings+upper extremity	2
adductors+upper extremity+psoas major	2
gastrocnemius+upper extremity+tibialis posterior	2
gastrocnemius+extensor hallucis longus	1
gastrocnemius+hamstrings+adductors+flexor hallucis longus,	1
gastrocnemius+flexor hallucis longus	1
gastrocnemius+adductors+flexor hallucis longus	1
gastrocnemius+hamstrings+flexor hallucis longus	1
gastrocnemius+hamstrings+adductors+flexor hallucis longus+psoas major+tibialis anterior	1
adductors+gluteus maximus	1
gastrocnemius+hamstrings+gluteus maximus	1
gracilis+rectus femoris	1

gracilis+rectus femoris+flexor hallucis longus+psoas major	1
hamstrings+psoas major	1
gastrocnemius+psoas major	1
hamstring+rectus femoris+gluteus maximus	1
gastrocnemius+hamstrings+rectus femoris	1
hamstrings+adductors+tibialis anterior	1
gastrocnemius+hamstrings+tibialis anterior	1
tibialis posterior+extensor hallucis longus	1
gastrocnemius +tibialis posterior+gracilis	1
gastrocnemius+hamstrings+tibialis posterior+gracilis	1
hamstrings+tibialis posterior+gracilis+psoas major	1
gastrocnemius+tibialis posterior+psoas major	1
gastrocnemius+tibialis posterior+rectus femoris+psoas major	1
hamstrings+adductors+tibialis posterior+rectus femoris+psoas major	1
hamstrings+adductors+upper extremity+flexor hallucis longus	1
gastrocnemius+hamstrings+adductors+upper extremity+flexor hallucis longus	1
hamstrings+adductors+upper extremity+flexor hallucis longus+psoas major+tibialis anterior	1
adductors+upper extremity+gluteus maximus	1
gastrocnemius+hamstrings+upper extremity+gracilis	1
hamstrings+adductors+upper extremity+psoas major	1
gastrocnemius+hamstrings+adductors+upper extremity+psoas major+tibialis anterior	1
adductors+upper extremity+rectus femoris	1
hamstrings+upper extremity+rectus femoris	1
adductors+upper extremity+tibialis posterior+flexor hallucis longus	1
gastrocnemius+hamstrings+upper extremity+tibialis posterior	1
gastrocnemius+hamstrings+adductors+upper extremity+tibialis posterior	1
missing data	25
